# 1616. Clinical Characteristics of Children with Congenital Cytomegalovirus Diagnosed Retrospectively by Dried Blood Spot

**DOI:** 10.1093/ofid/ofad500.1451

**Published:** 2023-11-27

**Authors:** Ana Del Valle Penella, Ryan H Rochat, Gail J Demmler-Harrison

**Affiliations:** Arkansas Children's Hospital / UAMS, Little Rock, Arkansas; Baylor College of Medicine, Houston, Texas; Baylor College of Medicine, Houston, Texas

## Abstract

**Background:**

Congenital cytomegalovirus (cCMV) is diagnosed by detection of CMV in saliva, urine, blood, or newborn dried blood spot (DBS) collected within the first 21 days of life (DOL).

**Methods:**

In this single-center retrospective study, all patients with a request for DBS testing between 2013 and 2021 were reviewed. We describe the clinical characteristics of children that were diagnosed with cCMV retrospectively via DBS at Texas Children’s Hospital.

DBS were requested from the Texas State Health Department and sent to the Center for Disease Control for testing by DNA CMV PCR.

Figure 1

**Figure:**
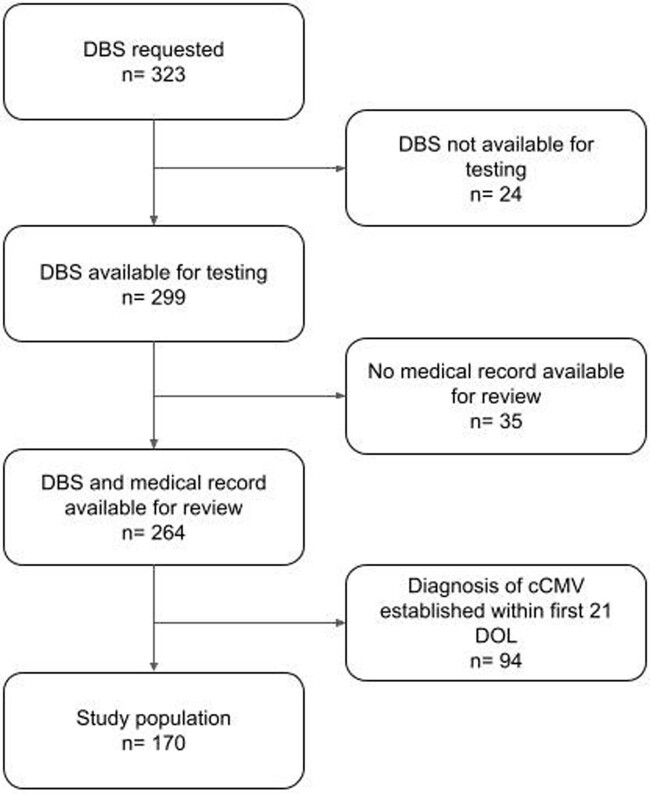

Inclusion and exclusion criteria of study population

**Results:**

DBS testing was done in 170 children with suspected cCMV. The median age of request for DBS testing was 3 months (IQR 2-6). The most common reason for testing was symptoms suggestive of cCMV with positive CMV testing outside 21 DOL (113/170, 66%). The most common symptom was sensorineural hearing loss (SNHL) (66/170, 39%), followed by developmental delays (28/170, 16%).

42 of 170 children (25%) had a positive DBS obtained within the first 21 DOL confirming the diagnosis of cCMV. 21/42 (51%) would have been classified as asymptomatic at birth, 11 (26%) as asymptomatic with isolated hearing loss, 4 (9%) as mild disease and 6 (14%) as moderate/severe disease.

Out of the 25 children with SNHL, 15 (60%) were congenital, 5 (20%) early onset and 5 were late onset (20%).

Of the 21 children diagnosed with cCMV via DBS that appeared asymptomatic at birth, only 3 (14%) remained asymptomatic at their last follow up. The most common symptom documented at follow up was learning delays (12/21, 50%) followed by SNHL (8/21, 38%). Brain imaging was obtained on 18 (86%) of them, and 10 (55%) had at least one abnormality identified that has been associated with cCMV.

40 children had at least one follow up visit at one year of age (median age of last follow up 3.5 years, range 1-9). 36 had at least one complication related to cCMV, most commonly, SNHL (24/36, 67%) and learning delays (24/36, 67%), cerebral palsy (9/36, 25%).

Table 1

**Figure:**
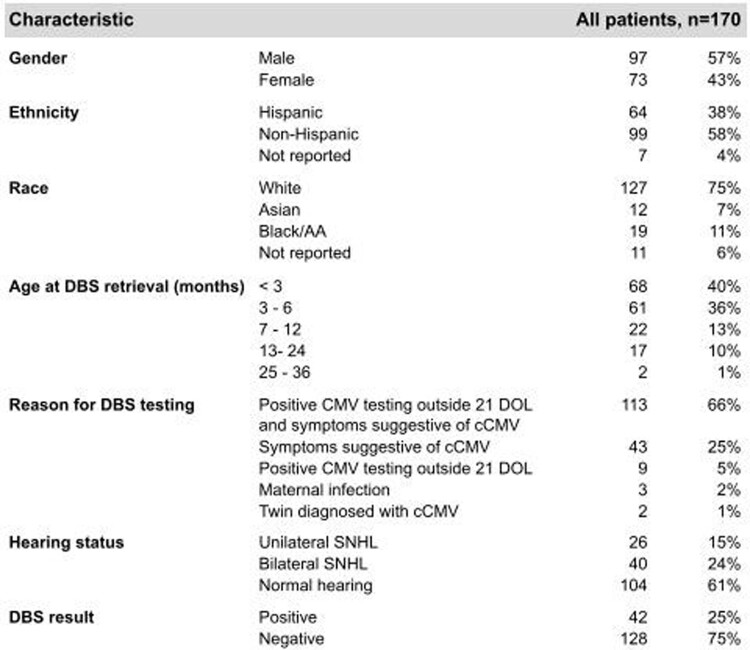

All children with suspected cCMV that underwent DBS testing

Table 2

**Figure:**
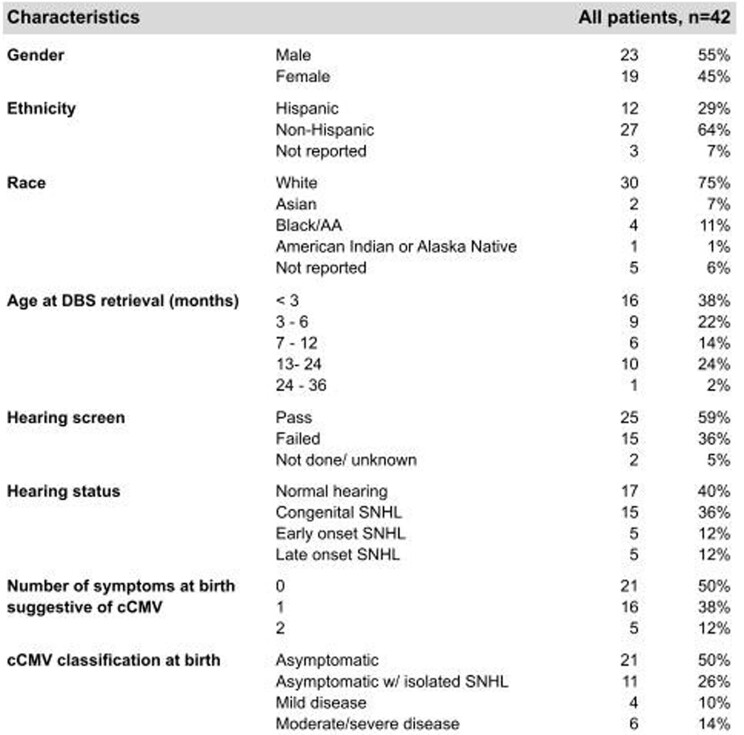

Characteristics of children diagnosed with cCMV via DBS retrospectively

Table 3

**Figure:**
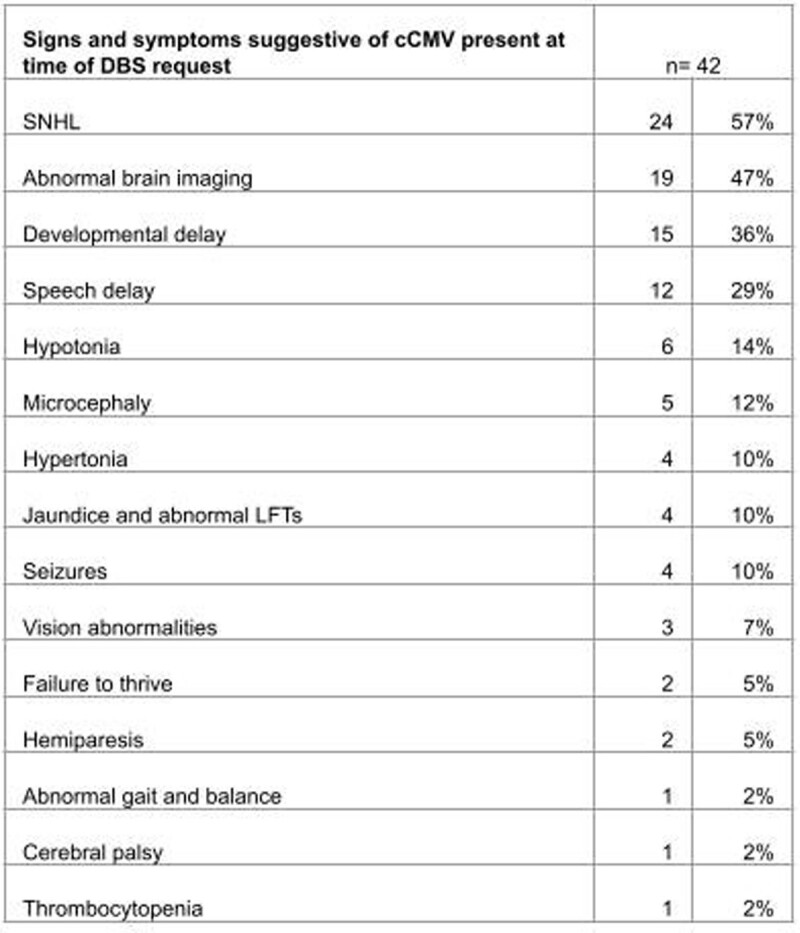

Signs and symptoms suggestive of cCMV present at time of DBS request in children diagnosed with cCMV via DBS

**Conclusion:**

Congenital CMV remains an underdiagnosed condition. Our study highlights the value of DBS for the diagnosis of cCMV, especially in asymptomatic newborns. Universal newborn screening will identify these infants early and allow for timely interventions, that may mitigate the development of long-term complications.

**Disclosures:**

**Gail J. Demmler-Harrison, MD**, Elsevier: Royalties for authorship/book production/editorship|Merck: Advisor/Consultant|Merck: Grant/Research Support|Microgen: Advisor/Consultant|Microgen: Grant/Research Support|Moderna: Advisor/Consultant|UpToDate Wolters Kluwer Health: Royalties for authorship Topics|WEBMD Medscape: Advisor/Consultant|WEBMD Medscape: Honoraria

